# Trimethylamine N-oxide Induced Chronic Kidney Injury by Triggering PANoptosis

**DOI:** 10.33549/physiolres.935576

**Published:** 2025-08-01

**Authors:** Decui SHAO, Lu BAI, Qian CHEN, Yuhong CHEN, Zhaoxu QIU, Yinuo LIU, Sheng JIN, Yuming WU, Jing DAI

**Affiliations:** 1 Laboratory of Cell Electrophysiology, Wannan Medical College, Anhui, China; 2 Department of Physiology, Hebei Medical University, Hebei, China; 3 Department of Critical Care Medicine, The Fourth Hospital of Hebei Medical University, Hebei, China; 4 Hebei Collaborative Innovation Center for Cardio-Cerebrovascular Disease, Hebei, China; 5 The Key Laboratory of Neural and Vascular Biology, Ministry of Education, Hebei, China; 6 Hebei Key Laboratory of Cardiovascular Homeostasis and Aging, Hebei, China; 7 Department of Clinical Diagnostics, Hebei Medical University, Hebei, China

**Keywords:** TMAO, Chronic kidney injury, Regulated cell death, PANoptosis

## Abstract

Trimethylamine N-oxide (TMAO) is involved in the development of kidney disease. However, the specific mechanism by which it leads to kidney injury is unclear. This study explored the role of regulated cell death in TMAO-induced kidney injury. We constructed a TMAO-induced chronic kidney injury model by intraperitoneal injection of TMAO (100 μmol/kg/day for three months). Plasma creatinine (Cre) and urea nitrogen (BUN) levels were measured to evaluate kidney function. Masson staining was used to evaluate kidney pathological changes. The expression levels of regulated cell death-related proteins were measured using western blotting. Plasma Cre and BUN, the area of kidney fibrosis in the TMAO group significantly increased. The western blotting results showed cleaved-Caspase-8, Caspase-8, Caspase-1, NOD-like receptor protein 3 (NLRP3), interleukin-1β (IL-1β), cleaved-gasdermin D (cleaved-GSDMD), Z-DNA binding protein 1 (ZBP1), phosphorylation of receptor-interacting protein kinase 3 (RIP3) and mixed-lineage kinase domain-like pseudokinase (MLKL) significantly elevated in the TMAO group. The transferrin receptor 1 (TFR1), ferritin heavy chain (FTH), ferroportin (FPN), nuclear factor erythroid 2-related factor 2 (NRF2), and glutathione peroxidase 4 (GPX4) protein expression in kidney tissue of the TMAO group significantly up-regulated. However, there was no change in iron and MDA levels. The results suggested that PANoptosis, including pyroptosis, apoptosis, and necroptosis components, might be involved in TMAO-induced chronic kidney injury.

## Introduction

Poor dietary habits, increasingly severe global climate change, population aging, and other factors have increased the burden on the kidneys, making kidney disease a hidden global epidemic. Its growth rate ranks third among the world’s causes of death, and it is expected to become the fifth-highest cause of death by 2040 [1–3]. Chronic kidney disease (CKD) is a common clinical syndrome secondary to many heterogeneous diseases that cause abnormalities in kidney structure and gradual kidney function impairment. Characterized by irreversibility and progressive evolution, CKD not only results in end-stage renal disease but also significantly increases risks of cardiovascular events, leading to increased mortality and health-care costs. Despite its irrefutable importance, its exact pathogenesis is not completely clarified [4–5].

In recent years, a growing body of evidence for the relationship between trimethylamine N-oxide (TMAO) and CKD have accumulated. TMAO is a metabolite product of choline, L-carnitine, and betaine. The unabsorbed choline and L-carnitine in the small intestine are metabolized by the gut microbiota to trimethylamine which is oxidized to TMAO in the liver by flavin monooxygenase 3 [6]. TMAO is mainly cleared from circulation by the kidneys, thus there is a strong inverse relationship between kidney function and circulating TMAO levels [7]. Elevated TMAO levels were closely linked with the degree of impaired kidney function in CKD and normalized by dialysis or kidney transplantation [8]. On the other hand, the accumulative TMAO also led directly to progressive kidney fibrosis and dysfunction, which might aggravate the CKD [9–10]. However, the exact underlying mechanisms by which TMAO induces kidney damage have not been well established.

CKD is characterized by a disorder in cell numbers and irreversible loss of nephrons, in which regulated cell death (RCD) pathways, including apoptosis, necroptosis, pyroptosis, and ferroptosis, play a central role [11]. PANoptosis is a novel form of inflammatory cell death which involves the interactions between pyroptosis, apoptosis, and necroptosis, but cannot be explained by any of them alone, and it also is related to the development of kidney diseases [12]. In addition, A growing body of evidence demonstrated that TMAO induced or aggravated multiple organ damage by activating the RCD pathways. Mechanistically, TMAO promoted CKD progression by inducing tubular ferroptosis and fibrosis [13]. Concurrently, TMAO impaired β-cell function and glucose tolerance through NLRP3 inflammasome-related cytokines [14]. Furthermore, TMAO also triggered premature ovarian insufficiency via the activation of mitochondrial pathway apoptosis in granulosa cells [15]. However, the potential link between TMAO-induced chronic kidney injury and PANoptosis remains elusive.

Therefore, the purpose of this study was to clarify whether PANoptosis was involved in TMAO-induced chronic kidney injury.

## Material and Methods

### Animals and treatments

Male 7-week-old C57BL/6J mice were purchased from Vital River Laboratories (Beijing, China) and housed in a standard raising condition with 12h light/12h dark cycles at 22 °C–24 °C and *ad libitum* access to food and water. All animal experiments were performed according to the Guide for the Care and Use of Laboratory Animals published by the US National Institutes of Health (NIH Publication, 8th Edition, 2011) and approved by the Ethics Committee for Laboratory Animals Care and Use of Hebei Medical University (IACUC-Hebmu-P2024072).

After acclimatization for one week, the mice were randomly divided into two groups: the Control group and the TMAO group. The mice in the TMAO group were intraperitoneally injected with TMAO (100 μmol/kg/day, Aladdin Biochemical Technology Co., Ltd., China) for three months, whereas the mice in the Control group were injected with normal saline for the same period. After three months, the mice were euthanized by intraperitoneally injecting an overdose of pentobarbital (100 mg/kg). After blood was collected, the kidney tissue samples were rapidly removed and fixed with 4 % paraformaldehyde or frozen at −80 °C until further analysis.

### Measurements of creatinine (Cre) and blood urea nitrogen (BUN) concentrations in plasma

Mouse blood samples were collected in EDTA anticoagulant tubes and immediately centrifuged at 1200 g for 15 min to obtain plasma. The plasma Cre levels were measured using Cre assay kits (Jiancheng Bioengineering Institute, China) by sarcosine oxidase enzymatic method. Briefly, the plasma was incubated with sequentially added reaction reagents at 37 °C for 5 min, twice in total, according to the manufacturer’s instructions. The absorbance was then measured twice at 546 nm, and the Cre content was calculated using two absorbances according to the formula. Plasma BUN levels were measured using BUN assay kits (Jiancheng Bioengineering Institute, China) by urease enzymatic method. Briefly, the plasma was incubated with sequentially added reaction reagents at 37 °C for 20 min and then absorbance was measured at 640 nm.

### Measurements of malondialdehyde (MDA) and iron levels in kidney tissue samples

After mechanical homogenization and centrifugation, the supernatant of kidney tissues was used to measure the MDA and iron concentrations by using commercial kits (Jiancheng Bioengineering Institute, China) according to the manufacturer’s instructions. The concentrations of MDA and iron were standardized by protein content, determined by a bicinchoninic acid protein assay kit (Beyotime Institute of Biotechnology, China).

### Masson’s trichrome analysis

After being fixed in 4 % paraformaldehyde for 48 h, the kidney tissues were routinely processed, embedded in paraffin, and sectioned into 5 μm-thickness slices. Masson’s trichrome staining was carried out to identify collagen deposition which was shown in blue and was quantified using ImageJ software (NIH, the United States). The semi-quantification was performed using the following formula: the collagen content = the area of Masson’s trichrome stained collagen (blue)/kidney area×100 %.

### Western blot analysis

Frozen kidney tissue samples (n=6 in each group) were homogenized with ice-cold RIPA lysis buffer. Total lysate protein concentrations were measured using a bicinchoninic acid protein assay kit (Beytime Institute of Biotechnology, China). Equal amounts of protein samples were separated on 10 % SDS-PAGE gels and transferred to polyvinylidene fluoride membranes. Non-specific binding to membranes was blocked by incubation with 5 % non-fat milk for 1 h. The blocked membranes were incubated with primary antibodies at 4 °C overnight and then incubated with horseradish peroxidase-conjugated secondary antibodies for 1 h after washing with TBST. Proteins were visualized with supersignal west pico chemiluminescent substrate (Thermo, the United States) and quantified using Image J software (NIH, the United States). The following primary antibodies were used: Caspase-8 (1:500, 1.3 μg/ml, Proteintech Biotechnology, the United States), Z-DNA binding protein 1 (ZBP1, 1:500, 1.6 μg/ml, Proteintech Biotechnology, the United States), mixed-lineage kinase domain-like pseudokinase (MLKL, 1:2000, 1 μg/ml, Proteintech Biotechnology, the United States), phosphorylation of MLKL (p-MLKL, 1:1000, 1.7 μg/ml, Abcam, the United States), receptor-interacting protein kinase 3 (RIP3, 1:1000, 0.6 μg/ml, Proteintech Biotechnology, the United States), phosphorylation of RIP3 (p-RIP3, 1:1000, 0.5 μg/ml, Abcam, the United States), Caspase-1 (1:1000, 0.65 μg/ml, Proteintech Biotechnology, the United States), NOD-like receptor protein 3 (NLRP3, 1:1000, 0.6 μg/ml, Proteintech Biotechnology, the United States), cleaved-gasdermin D (cleaved-GSDMD, 1:1000, 0.1 μg/ml, Cell Signaling Technology, Inc., the United States), interleukin-1β (IL-1β, 1:1000, 0.7 μg/ml, Proteintech Biotechnology, the United States), nuclear factor erythroid 2-related factor 2 (NRF2, 1:1000, 0.7 μg/ml, Proteintech Biotechnology, the United States), glutathione peroxidase 4 (GPX4, 1:1000, 1 μg/ml, Proteintech Biotechnology, the United States), ferroportin (FPN, 1:2000, 0.3 μg/ml, Proteintech Biotechnology, the United States), ferritin heavy chain (FTH, 1:2000, 0.5 μg/ml, Immunoway, the United States), transferrin receptor 1 (TFR1, 1:1000, 0.69 μg/ml, Abcam, the United States), GAPDH (1:5000, 0.12 μg/ml, Proteintech Biotechnology, the United States), β-actin (1:2000, 0.2 μg/ml, Proteintech Biotechnology, the United States) and β-Tubulin (1:5000, 0.12 μg/ml, Proteintech Biotechnology, the United States).

### Statistical Analysis

Statistical analysis was performed using SPSS software package (SPSS 17.0, Inc., the United States). Data were expressed as mean ± SEM and analyzed using an independent t-test when comparing between two groups. P<0.05 was considered statistically significant.

## Results

### TMAO induced chronic kidney injury

**Fig. 1:**
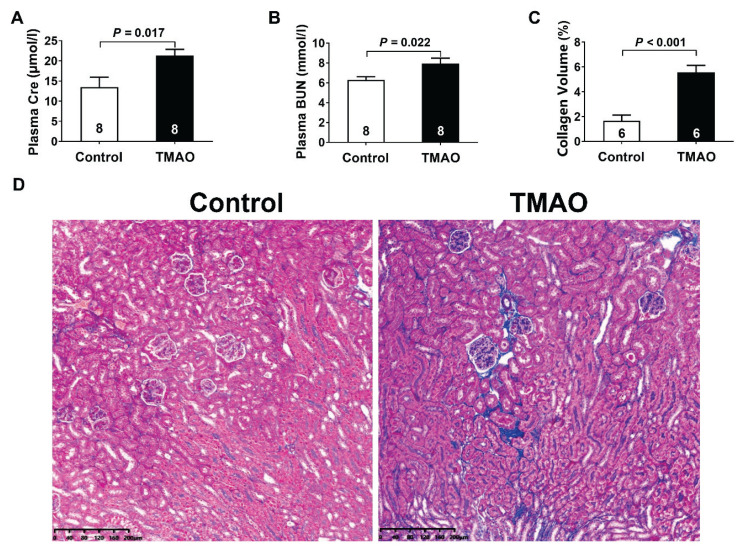
TMAO induced chronic kidney injury. (**A**) Plasma creatinine (Cre) levels. (**B**) Plasma blood urea nitrogen (BUN) levels. (**C**) The quantitative analysis for collagen volume fraction (%) in kidney tissues. (**D**) Representative Masson’s trichrome-stained kidney sections. Results are expressed as mean ± SEM. A *P* of <0.05 was considered significant.

As shown in Figure 1, compared with the control group, TMAO-treated mice demonstrated significant kidney dysfunction characterized by 58.18 % and 25.93 % increases in plasma Cre and BUN levels, respectively. Histological analysis through Masson’s trichrome staining revealed notable kidney fibrosis, with collagen deposition area expanding from 1.67 % in control group to 5.58 % in TMAO group. This three-fold augmentation of fibrotic lesions, coupled with deteriorated kidney function biomarkers, strongly indicated that TMAO exposure for three months induced chronic kidney injury and fibrosis.

### Apoptosis was involved in TMAO-induced chronic kidney injury

**Fig. 2:**
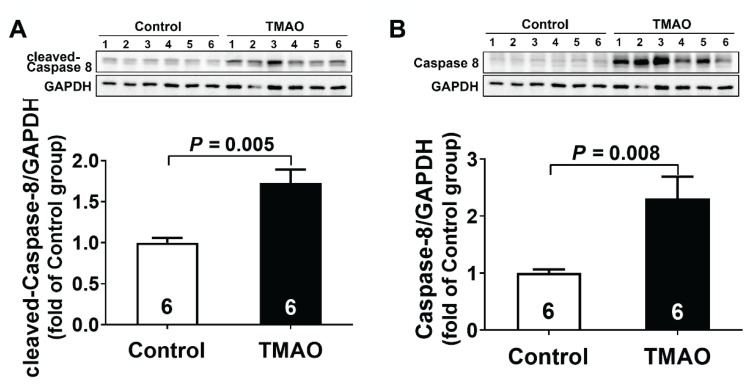
Apoptosis was involved in TMAO-induced chronic kidney injury. (**A–B**) Representative western blots and quantitative analysis for cleaved-Caspase 8 and Caspase-8 protein expression in kidney tissues after TMAO treatment. Results are expressed as mean ± SEM. A *P* of <0.05 was considered significant.

As shown in Figure 2, TMAO administration for three months significantly upregulated both the precursor and activated forms of Caspase-8 protein expression in kidney tissues, with cleaved Caspase-8 levels increasing by 1.7-fold and total Caspase-8 expression rising by 2.3-fold compared to control group. This quantitative evidence confirmed the activation of extrinsic apoptotic signaling, directly linking Caspase-8-mediated apoptosis to TMAO-induced chronic kidney injury.

### Necroptosis mediated TMAO-induced chronic kidney injury

**Fig. 3:**
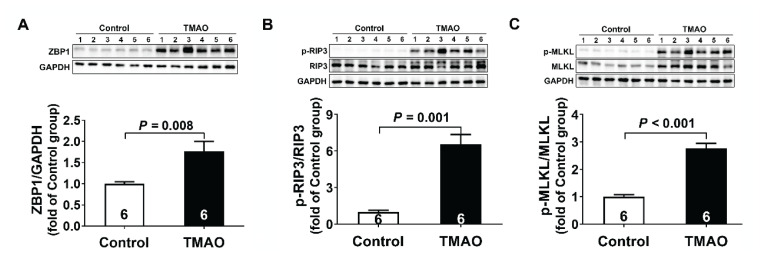
Necroptosis mediated TMAO-induced chronic kidney injury. (**A–C**) Representative western blots and quantitative analysis for ZBP1, p-RIP3/RIP3 and p-MLKL/MLKL protein expression in kidney tissues after TMAO treatment. Results are expressed as mean ± SEM. A P of <0.05 was considered significant.

As shown in Figure 3, TMAO treatment for three months significantly up-regulated the protein expression levels of ZBP1, accompanied by enhanced phosphorylation of RIP3 and MLKL as compared to the control group. Quantification revealed 1.8-, 2.3-, and 6.5-fold increases in ZBP1, p-RIP3, and p-MLKL levels respectively. The sequential activation of ZBP1-RIP3-MLKL axis demonstrated ZBP1-mediated necroptosis signaling pathway was involved in TMAO-induced chronic kidney injury.

### Pyroptosis mediated TMAO-induced chronic kidney injury

**Fig. 4:**
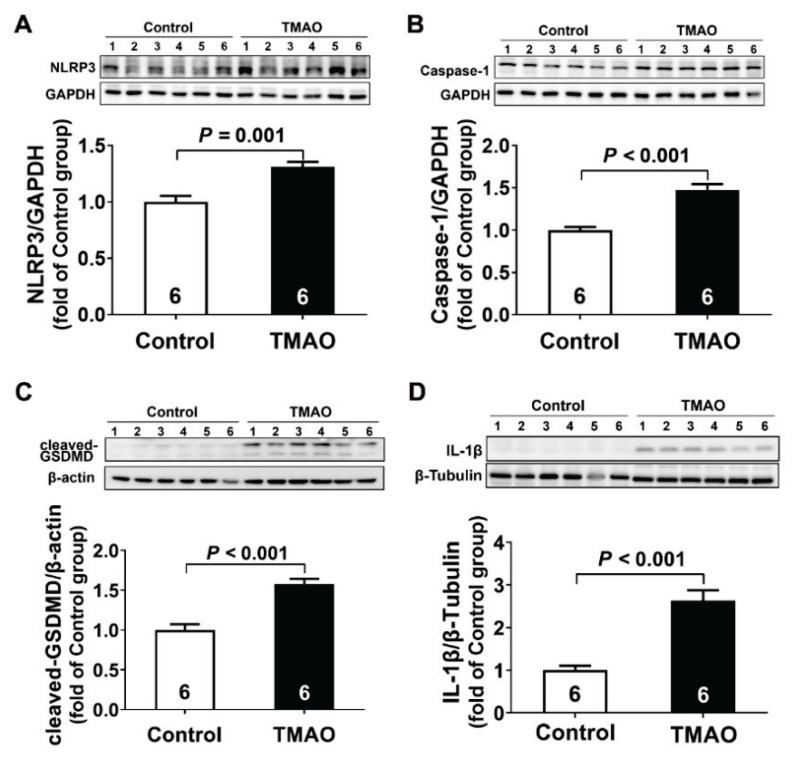
Pyroptosis mediated TMAO-induced chronic kidney injury. (**A–D**) Representative western blots and quantitative analysis for NLRP3, Caspase-1, cleaved-GSDMD, and IL-1β protein expression in kidney tissues after TMAO treatment. Results are expressed as mean ± SEM. A *P* of <0.05 was considered significant.

To determine whether the NLRP3 inflammasomes was activated by TMAO, we investigated the expression of NLRP3 and Caspase-1, which were considered accurate indicators of inflammasome activation. As shown in Figure 4A and 4B, TMAO promoted NLRP3 and Caspase-1 expression by 1.3-fold and 1.5-fold respectively in kidney tissue as compared to the control group. Given NLRP3 inflammasome activation was closely related to pyroptosis, we further investigated whether pyroptosis was occurred after TMAO treatment. As presented in Figure 4C and 4D, TMAO also induced a marked increase in cleaved-GSDMD (1.6-fold), and IL-1β (2.9-fold) protein expression. The collective results suggested that NLRP3/caspase-1/GSDMD-mediated pyroptosis was activated in TMAO-induced chronic kidney injury.

### Ferroptosis was not involved in TMAO-induced chronic kidney injury

**Fig. 5:**
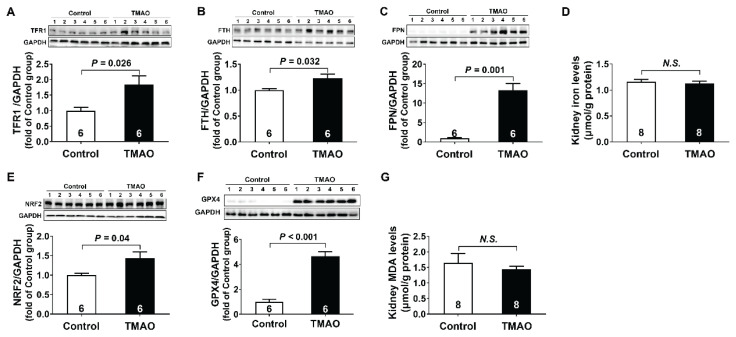
Ferroptosis was not involved in TMAO-induced chronic kidney injury. (**A–C**) Representative western blots and quantitative analysis for TFR1, FTH, and FPN protein expression in kidney tissues after TMAO treatment. (**D**) Iron levels in kidney tissues after TMAO treatment. (**E–F**) Representative western blots and quantitative analysis for NRF2 and GPX4 protein expression in kidney tissues after TMAO treatment. (**G**) MDA levels in kidney tissues after TMAO treatment. Results are expressed as mean ± SEM. A *P* of <0.

To explore whether ferroptosis was involved in TMAO-induced chronic kidney injury, ferroptosis-related molecules such as TFR1, FTH, FPN, NRF2, and GPX4 protein expression in kidney tissue were assessed. As demonstrated in Figure 5A–5C, TMAO administration induced significant elevation of iron metabolism-related proteins compared to controls, including TFR1 (1.8-fold), FTH (1.2-fold), and FPN (13.3-fold), while the concentrations of iron in the kidney did not change (Fig. 5D). In addition to abnormal iron metabolism, the maladjustment of redox systems is another main characteristic of ferroptosis. As shown in Figure 5E–5F, compared with the control group, the TMAO group showed significant up-regulation of NRF2 (1.4-fold) and GPX4 (4.9-fold) protein, the key transcription factor and antioxidant enzyme that regulated cellular oxidative stress. However, there was no difference in the concentrations of MDA in kidney tissue between the two groups. These results indicated that ferroptosis was not related to TMAO-induced chronic kidney injury.

## Discussion

In this study, we found that TMAO directly damages the kidneys and PANoptosis, including apoptosis, necroptosis, and pyroptosis, was involved in TMAO-induced chronic kidney injury. In contrast, ferroptosis was not involved in this injury.

TMAO, a gut microbiota-derived metabolite, is derived from precursor choline, phosphatidylcholine, betaine, and carnitine in dietary nutrients and primarily eliminated via kidney excretion. Accumulating evidence showed that the elevated levels of TMAO was not only a key biomarker but also a direct causative factor for a wide variety of human diseases, including CKD [8,16]. It was reported that TMAO directly led to progressive kidney function decline and fibrosis [17], while inhibition of TMAO production attenuated CKD development [18]. In addition, TMAO also directly induced cardiac dysfunction and fibrosis [19–20]. In line with these studies, we found that plasma Cre and BUN levels, indicators of kidney function, were significantly increased in mice treated with TMAO and Masson staining analysis showed that the fibrotic area caused by TMAO treatment was more significant than that of the control group. The above results clarified that TMAO exposure for three months directly caused chronic kidney injury and fibrosis.

Emerging evidence highlighted the pivotal role of RCD in CKD pathogenesis, while RCD also contributed to TMAO-induced organ injury. To bridge these findings, the present study systematically investigated the contributions of multiple RCD pathways, such as ferroptosis, apoptosis, necroptosis, and pyroptosis, in TMAO-induced chronic kidney injury.

Ferroptosis is defined as an iron-dependent form of RCD driven by two major molecular mechanisms, including iron overload originally from abnormal iron metabolism and maladjustment of redox systems [21,22]. Recent reports have implicated ferroptosis in multiple diseases, including kidney diseases [23,24]. In the present study, although the expression of ferroptosis-related molecules, such as TFR1, FTH, FPN, NRF2, and GPX4 in kidney tissue was significantly up-regulated, there was no significant increase in iron and MDA (a biomarker of oxidative stress) contents after three months of TMAO treatment. During the early pathological phase, TMAO might induce upregulation of TFR1, a key iron uptake protein, thereby enhancing cellular iron influx and triggering oxidative stress. Subsequently, the FTH (an iron storage protein), FPN (an iron export transporter), NRF2 (a transcription factor to counteract oxidative stress), and GPX4 (an antioxidant enzyme) were compensatory up-regulated to reduce intracellular iron and oxidative damage. These results indicated that ferroptosis was not related to TMAO-induced chronic kidney injury. Consistent with our findings, ferroptosis is more prominently implicated in acute kidney injury than in CKD [25].

Apoptosis is the most well-characterized form of RCD to maintain organismal homeostasis triggered through two major pathways referred to as the intrinsic and extrinsic pathways [26]. We found that TMAO administration for three months significantly upregulated both the precursor and activated forms of Caspase-8 protein expression in kidney tissues, indicating the occurrence of extrinsic apoptosis. In line with our study, it was reported that TMAO promoted hyperoxaluria-induced calcium oxalate deposition and kidney injury by activating apoptosis and autophagy signal pathway [27], meanwhile, inhibition of TMAO-induced apoptosis could reduce kidney damage and fibrosis [28].

Pyroptosis and necroptosis are two lytic, inflammatory types of RCD that are triggered by inflammasomes and necrosome and executed through the membrane damaging GSDMD and MLKL proteins, respectively ^[29]^. In the present study, we found that TMAO treatment significantly upregulated NLRP3, Caspase-1, cleaved-GSDMD, and IL-1β protein expression in kidney tissue as compared to the control group, indicating NLRP3/caspase-1/GSDMD-mediated pyroptosis was activated in TMAO-induced chronic kidney injury. Meanwhile, TMAO treatment also upregulated ZBP1 protein expression and phosphorylation of RIP3 and MLKL, suggesting that ZBP1-mediated necroptosis signaling pathway was involved in. To date, while there have been no reports of TMAO causing necroptosis, emerging evidence on TMAO-induced pyroptosis aligned closely with our findings. It was reported that TMAO promoted diabetic kidney disease by activating NLRP3 inflammasome to induce pyroptosis [30]. Conversely, inhibition of TMAO attenuated neointimal formation through reduction of NLRP3 inflammasome in a mouse model of carotid artery ligation [31]. TMAO also aggravated graft versus host disease by activating NLRP3 inflammasome to polarize M1 macrophage [32].

Earlier research highlighted the unique regulation in each of RCD pathways, but emerging studies discovered co-regulation and crosstalk between these seemingly different cell death complexes. To address this complexity, a new, comprehensive form of cell death that encompasses apoptosis, necroptosis, and pyroptosis, has recently been described, termed PANoptosis which is driven by caspases and RIPKs and regulated by multiprotein PANoptosome complexes ^[33]^. To date, four types of PANoptosome have been identified, including ZBP1-regulated, AIM2-regulated, RIPK1-regulated, and NLRP12-regulated PANoptosome, among which ZBP1-regulated PANoptosome were the first to be discovered [34–35]. The ZBP1 PANoptosome can engage, in parallel, pyroptosis, apoptosis, and necroptosis and implicates in a wide array of human diseases ^[36–37]^. In the present study, we found that TMAO exposure triggered coordinated activation of ZBP1-mediated PANoptosome signaling, including caspase-8-dependent apoptosis, NLRP3-driven pyroptosis, MLKL-executed necroptosis, but notably absent of ferroptosis, suggesting that ZBP1-PANoptosis existed in TMAO-induced chronic kidney injury, however, the precise regulatory crosstalk between these pathways requires further mechanistic investigation.

There are several limitations in the current study. Firstly, in present study, TMAO was intraperitoneally injected for three months to induce chronic kidney injury, however, the systemic effects of TMAO on non-renal organs, such as cardiovascular system, were not comprehensively assessed. In addition, the potential effect of TMAO on chronic kidney injury through multi-organ interaction was not considered. Therefore, future work should employ systems biology approaches to map inter-organ signaling networks under TMAO stress. Secondly, this study found that PANoptosis was involved in TMAO-induced chronic kidney injury, however, the molecular mechanism by which TMAO activated PANoptosis, such as potential transcriptional regulators or post-translational modifications, and the specific molecular targets of TMAO required further study. Finally, to conclusively establish the therapeutic potential of TMAO, inhibitors of TMAO or PANoptosis should be applied in future studies to validate its pathogenic role and delineate downstream molecular mechanisms.

In summary, the present study suggested that PANoptosis was involved in TMAO-induced chronic kidney injury and developing targeted drugs based on our experiments will be beneficial for the treatment of kidney diseases. However, much work was still needed to understand the mechanism of PANoptosis and to provide more suitable targets for the treatment of TMAO-induced chronic kidney injury.
